# Conversion of oleuropein to hydroxytyrosol by lactic acid bacteria fermentation of olive leaves in water solution with reduced glucose content

**DOI:** 10.1007/s11274-025-04495-4

**Published:** 2025-07-28

**Authors:** Zuzana Farkas, Rosa Romeo, Domenico Pangallo, Lucia Kraková, Angelo M. Giuffrè, Rossana Sidari

**Affiliations:** 1 Institute of Molecular Biology, Slovak Academy of Science, Dúbravská cesta 21, Bratislava, 84551 Slovakia; 2 Department of Agraria, Mediterranea University of Reggio Calabria, Reggio Calabria, 89122 Italy; 3 Department of Biology, Institute of Biology and Biotechnology, Faculty of Natural Sciences, University of SS. Cyril and Methodius, Námestie Jozefa Herdu 2, Trnava, 91701 Slovakia

**Keywords:** Acidic pH, Bioconversion, Glucose, Hydroxytyrosol, Lactic acid bacteria, Oleuropein, Olive leaves

## Abstract

Oleuropein is the most abundant bioactive phenolic compound olive trees (*Olea europaea* L.). It is found in all parts of the plant, but especially in the leaves. This study describes the bioconversion of oleuropein to hydroxytyrosol, a polyphenol with antioxidant and antibacterial properties, by the fermentation of olive leaves by lactic acid bacteria (LAB), using a new, more eco-friendly method that is not based on chemical solvent extraction. This method uses an aqueous solution with reduced glucose content to which ground leaves are added and subsequently inoculated with LAB strains. In this experiment, the pH, glucose, and LAB strains are key factors. We tested a total of fourteen LAB strains for *β*-glucosidase activity, from which we selected the five with the best demonstrated activity - *Lactiplantibacillus plantarum* PB22, *Fructilactobacillus sanfranciscensis* B415, *Lactiplantibacillus pentosus* B506, *Lactiplantibacillus pentosus* B307 and *Lactiplantibacillus plantarum* B329. The bioconversion was monitored over 28 days using a UPLC system coupled with a UV/Vis Photo Diode Array. The best strains for converting oleuropein to hydroxytyrosol were *F. sanfranciscensis* B415, *L. pentosus* B506, and *L. pentosus* B307 and the optimum fermentation time was found to be 3 days. This work proposes an environmentally friendly low-impact method for reusing agricultural plant wastes.

## Introduction

The olive tree (*Olea europaea* L.) is a plant widely cultivated in Mediterranean countries for the production of olives and olive oil, which are rich in health-promoting compounds, including polyphenols. Their polyphenolic content was found to be influenced by many parameters including the olive cultivar (Sicari et al. 2021) and the degree of olive fruit ripening (Giuffrè 2018). Also, the olive leaves contain polyphenols such as oleuropein – one of a group of coumarin-like compounds, called secoiridoids, which are abundant in *Oleaceae* – as well as hydroxytyrosol (3,4-(dihydroxyphenyl)ethanol), tyrosol, rutin, cumaric acid, ferulic acid, caffeic acid, etc. (Soler-Rivas et al. 2000). The concentration of oleuropein and hydroxytyrosol in olive leaves is 60–90 mg g^− 1^ and 0.78–136.48 mg g^− 1^, respectively (Soler-Rivas et al. 2000; Martínez-Navarro et al. 2023). These compounds have antioxidant activity and play a role in protection from cardiovascular diseases, non-alcoholic fatty liver diseases, oxidative stresses, neurodegenerative diseases, and cancer (Somova et al. 2003; Skerget et al. 2005; Khayyal et al. 2002; Abenavoli et al. 2019; Markhali et al. 2020; Cerri et al. 2024; Wang et al. 2025a). In particular, hydroxytyrosol has anti-inflammatory, anti-cancer, antiviral, and antimicrobial activities and also improves endothelial dysfunction, decreases oxidative stress, and protects the nervous and cardiovascular systems (Bertelli et al. 2020). As for the olives, the pre- and post-harvest conditions of the leaves affect the concentration and functional properties of their phenolic compounds (Markhali et al. 2020).

The olive leaves are a waste product derived by the pruning of trees and the harvesting of olives (Sorgonà et al. 2018); in fact, they can account for as much as 10% of the total weight of processed olives. Managing them can increase production costs (Bouaziz et al. 2008), and they have to be properly disposed of to avoid environmental problems (Romero-García et al. 2014). Presently, there is great interest in re-using this waste by converting it into valuable compounds with potential applications in pharmaceuticals, cosmetics, feeds and functional foods while also contributing to a circular and green economy (Souilem et al. 2017; Markhali et al. 2020). One potential approach involves the extraction of oleuropein from them and its bioconversion to hydroxytyrosol. This process proceeds through the hydrolysis of oleuropein into an aglycone and its subsequent hydrolysis into one of two forms of elenoic acid and hydroxytyrosol (Briante et al. 2002). Other bioactive compounds may also be produced.

Some reports have described the processing of the leaves by physical, enzymatic or microbiological methods to produce polyphenols for use as human dietary supplements or feedstuff additives (Al-Azzawiea and Alhamdanib 2006; Xie et al. 2015, 2016; Yuan et al. 2015). Currently, microbiological olive leaf treatments employ fungi such as *Aspergillus niger*, *Aspergillus oryzae*, *Trichoderma viride*, *Candida utilis*, *Candida tropicalis* and *Geotrichum candidum* (Xie et al. 2016; Altop 2019) while lactic acid bacteria (LAB) are mainly used for treating the olive pomace and the olive mill wastewater in order to obtain hydroxytyrosol from oleuropein (Papadaki and Mantzouridou 2016; Iorizzo et al. 2016; Romeo et al. 2021). Recent studies have described the solid-state fermentation of olive leaves with *Lactobacillus acidophilus* in co-culture with *A. niger*, *A. oryzae* and *C. utilis* (Sar et al. 2024), the fermentation of olive leaf brine with *Lactobacillus plantarum*, and olive leaf fermentation with *Lactobacillus lactis* (Ilgaz et al. 2023; Bouter et al. 2012). These studies do make use of LAB, along with other microorganisms, to obtain bioactive compounds from olive leaves, but the processes reported involved the use of either chemical solvents or physical methods to improve the extraction of the bioactive compounds. The current study fills a gap in the literature by describing the development of a bioconversion method using LAB that does not involve the use of chemical solvents or additional instruments.

The conversion of oleuropein to hydroxytyrosol is mainly due to β-glucosidases, a class of enzymes present in many different microorganisms, including LAB (Gueguen et al. 1997; Sestelo et al. 2004; Michlmayr and Kneifel 2014). The ability of *Lactiplantibacillus plantarum* (formerly *Lactobacillus plantarum*) to degrade oleuropein has been described (Marsilio and Lanza 1998; Aponte et al. 2018; Romeo et al. 2021) together with that of other species, such as *Lactiplantibacillus pentosus*, *Lacticaseibacillus casei* and *Lacticaseibacillus paracasei*, *Levilactobacillus brevis* (Santos et al. 2012; Papadaki and Mantzouridou 2016; Iorizzo et al. 2016; Michlmayr et al. 2010).

The aim of this work was to screen fourteen LAB strains and select those able to convert oleuropein to hydroxytyrosol when in contact with ground olive leaves. In order to develop a simple, eco-friendly, cost-effective microbiological method for obtaining hydroxytyrosol.

## Materials and methods

### Bacterial strains and storage conditions

Fourteen LAB strains from the Microbial collection of the Department of Agraria (*Mediterranea* University of Reggio Calabria, Italy) were used in this study: *Lactiplantibacillus plantarum* PB1 (Zheng et al. 2020) (formerly *Lactobacillus plantarum*), *Lactiplantibacillus plantarum* PB22, *Lactiplantibacillus plantarum* PB40, *Lactiplantibacillus plantarum* PB61, *Lactiplantibacillus plantarum* PB70, *Lactiplantibacillus plantarum* B167, *Lactiplantibacillus plantarum* B283, *Lactiplantibacillus pentosus* B307 (Zheng et al. 2020) (formerly *Lactobacillus pentosus*), *Lactiplantibacillus plantarum* B329, *Lactiplantibacillus pentosus* B366, *Fructilactobacillus sanfranciscensis* B415 (Zheng et al. 2020) (formerly *Lactobacillus sanfranciscensis*), *Latilactobacillus sakei* B434 (Zheng et al. 2020) (formerly *Lactobacillus sakei*), *Lactiplantibacillus pentosus* B506, and *Latilactobacillus curvatus* B515 (Zheng et al. 2020) (formerly *Lactobacillus curvatus*). The strains were maintained at − 80 °C using cryobeads (Microbank TM, Pro-Lab Diagnostics, Canada) until use.

### Petri plate assay for screening the β-glucosidase activity of LAB strains

The LAB strains were tested for β-glucosidase activity (β-glu) using a qualitative screening on a chromogenic substrate according to Lorn et al. (2021) but with arbutin instead of esculin. The Arbutin Iron Agar (AIA) contained meat extract (3 g L^− 1^), peptone (5 g L^− 1^), agar, (16 g L^− 1^), arbutin (1 g L^− 1^), and iron-ammonium citrate (0.5 g L^− 1^).

A cryobead of each strain was transferred from frozen storage in tubes containing 10 mL of De Man Rogosa and Sharpe broth (MRS; VWR, Geldenaaksebaan, Leuven, Belgium) and incubated statically overnight at 30 °C. The strain cultures were then standardized to obtain an initial optical density OD_600nm_ = 1.0. Subsequently, 5 µL of each pre-culture was spotted in Petri plates containing AIA medium to detect β-glu activity. The plates were incubated for 3 days at 30 °C. After incubation, the LAB strains with the highest β-glu activity (those which changed the color of the medium from light yellow to dark brown) were selected for further experiments.

### Collection and processing of olive leaf samples

Olive leaf samples were collected during the winter months from olive trees (variety *Geracese*) located in the Bivongi village in the province of Reggio Calabria, Calabria (South Italy). First, the leaves were stripped of their stems, washed with 70% aqueous ethanol and dried at room temperature. Then, the leaves were finely ground using a sterilized laboratory mill and stored at − 20 °C until analyzed.

### Bioconversion of oleuropein to hydroxytyrosol by LAB

The following LAB strains were chosen following β-glu screening: *Lactiplantibacillus plantarum* PB22 (previously isolated from orange peel) (Calabrò et al. 2020), *Fructilactobacillus sanfranciscensis* B415 and *Lactiplantibacillus pentosus* B506 (isolated from sourdough) (Martorana et al. 2018), and *Lactiplantibacillus pentosus* B307 and *Lactiplantibacillus plantarum* B329 (both isolated from fermented olives). These LAB strains, maintained at − 80 °C, were cultured overnight in MRS broth at 30 °C before use. The strains PB22, B415, B506 and B307 were identified by the sequencing and multiplex PCR of the *recA* gene (see references above) while the strain B329 was identified by multiplex PCR of the *recA* gene (Torriani et al. 2001) in comparison with the reference strains *Lactobacillus plantarum* subsp. *plantarum* LMG 06907^T^ (BCCM/LMG Bacteria Collection, Laboratorium voor Microbiologie, Universiteit Gent, Belgium), *Lactobacillus paraplantarum* LMG 16,673^T^, and *Lactobacillus pentosus* LMG 10,755^T^.

Oleuropein, the most representative polyphenolic constituent of olive leaves (Le Toutour and Guedon 1992; Yateem et al. 2014), was extracted from the leaves and hydrolyzed to hydroxytyrosol by the β-glu activity of the LAB strains during a four week experiment. Ten grams of ground olive leaves were placed into a sterile Erlenmeyer flask containing 100 mL of MRS broth with reduced glucose content (0.3 g L^− 1^) (Marsilio and Lanza 1998) and inoculated with each chosen LAB strain (PB22, B307, B329, B415, B506). This culture was grown overnight and standardized to an initial OD_600nm_ = 1. The trials were statically incubated at 30 °C for 28 days and samples were collected for chemical analyses after 3, 7, 14, 21, and 28 days. Un-inoculated MRS broth with ground olive leaves served as a control. All experiments were performed in duplicate biological replicates.

### Analyses and identification of oleuropein and hydroxytyrosol

The pH of the samples collected after 3, 7, 14, 21 and 28 days was measured (Crison basic 20, Hach Lange Spain, S.L.U.) and the amounts of oleuropein and hydroxytyrosol present were determined using a UPLC system (PLATINblue, Knauer, Germany) coupled with a UV/Vis Photo Diode Array. Each 5 µL sample was centrifuged at 12,000 rpm for 10 min, filtered through a 0.45 μm cellulose membrane (Millipore-Interface; Amadora, Portugal), injected into the machine and analyzed following the method reported by De Bruno et al. (2022) with some modifications. A C18 column (Knauer, 1.8 μm, 150 × 3 mm) maintained at 30 °C with a flow rate of 0.4 mL min^− 1^ was used. The mobile phase was 0.1% acetic acid in water (A) versus acetonitrile (B) and the total running time was 20 min. The gradient changed as follows: 95% A (0–3 min); 95–60% A (3–15 min); and 60–0% A (15–20 min). Oleuropein and hydroxytyrosol quantification was performed using external standards (1–100 mg L^− 1^) and results were expressed as mg L^− 1^. The standards were oleuropein (99%), CAS 32619-42-4, from Extrasynthèse (Z.I. Lyon Nord, France) and 3-hydroxytyrosol (> 98%), CAS 10597-60-1, from TCI (Saitama, Japan). After 28 days, the LAB strain concentration of each trial was analyzed using a standard method in Petri plates containing MRS agar. The plates were incubated under anaerobic conditions at 30 °C for 48 h.

### Statistical analyses

Data were analyzed by a one-way ANOVA and a Tukey’s test, at 5% probability, using SPSS 17.0 (SPSS Inc., Chicago, IL, USA).

## Results

### Detection of β-glucosidase activity in LAB strains

*β*-glucosidase is the enzyme mainly responsible for hydrolyzing oleuropein, therefore, the *β*-glu activity of the individual LAB strains was qualitatively assessed on arbutin rich AIA media in Petri dishes. Fourteen strains were tested, five of which (PB22, B307, B329, B415, B506) gave a positive result, defined here as turning the medium brown (Table 1). *L. pentosus* B307, *F. sanfranciscensis* B415, and *L. pentosus* B506 had the highest activity. These strains were then used in the main trial involving olive leaves.

**Table 1:** β-glucosidase activity of LAB strains tested on AIA medium

Strain	β-glucosidase activity^a^	
*Lactiplantibacillus plantarum* PB1	-	
*Lactiplantibacillus plantarum* PB22	+	
*Lactiplantibacillus plantarum* PB40	-	
*Lactiplantibacillus plantarum* PB61	-	
*Lactiplantibacillus plantarum* PB70	-	
*Lactiplantibacillus plantarum* B167	-	
*Lactiplantibacillus plantarum* B283	-	
*Lactiplantibacillus pentosus* B307	+++	
*Lactiplantibacillus plantarum* B329	++	
*Lactiplantibacillus pentosus* B366	-	
*Fructilactobacillus sanfranciscensis* B415	+++	
*Latilactobacillus sakei* B434	-	
*Lactiplantibacillus pentosus* B506	+++	
*Latilactobacillus curvatus* B515	-	

^a^Activity: based on the color of the medium: -, no colour change; +, very light brown; ++, light brown; +++, brown

### pH and LAB count

LAB are well known to rapidly reduce the pH of the medium to the point where competing microorganisms are no longer able to survive (Steinkraus 1992). The pH values measured throughout the experiment (3–28 days) decreased relative to the control (pH 5.94) in all trial cultures inoculated with LAB strains with only slight differences observed between them (Table 2). Strain B506 (*L. pentosus*) showed the greatest acidifying tendency, with a reduction in pH from the beginning of the experiment by 1.73 units relative to the control with the other strains close behind.

**Table 2:** pH of the Olive leaf broth after fermentation with the given LAB strains

Analysis time (days)	pH					
	B415	B506	B307	PB22	B329	**Sign**
3	4.32 ± 0.01^bA^	4.29 ± 0.01^dB^	4.30 ± 0.01^cC^	4.40 ± 0.01^bB^	4.30 ± 0.01^bA^	**
7	4.26 ± 0.01^cA^	4.23 ± 0.01^eC^	4.29 ± 0.01^dE^	4.35 ± 0.01^bB^	4.32 ± 0.01^cD^	**
14	4.21 ± 0.01^abA^	4.13 ± 0.01^cA^	4.19 ± 0.00^cC^	4.26 ± 0.01^aB^	4.32 ± 0.01^bA^	**
21	4.26 ± 0.01^aA^	4.26 ± 0.00^aB^	4.25 ± 0.00^bC^	4.35 ± 0.01^bB^	4.25 ± 0.01^aA^	**
28	4.30 ± 0.01^bA^	4.21 ± 0.01^bA^	4.29 ± 0.01^aA^	4.38 ± 0.01^bB^	4.31 ± 0.02^bA^	**
**Sign**	**	**	**	**	**	

The data are presented as means ± SDs. **Significance at *P* < 0.01. By Tukey’s multiple range test, small letters show differences during the time for all samples and capital letters show differences among the samples at the same time

At the end of 28 days, the load of the LAB strains was measured. Table 3 shows the LAB load expressed as Log CFU/mL. *L. plantarum* PB22, *L. pentosus* B307, and *L. pentosus* B506 had the highest counts followed by *L. plantarum* B329 and *F. sanfranciscensis* B415 which had a load almost 1 log less. All strains maintained a high concentration until the end of the experiment (28 days), demonstrating that olive leaves are able to support their growth in low-glucose medium.

**Table 3:** LAB strain load after 28 days of Olive leaf fermentation

LAB strain	Log CFU/mL
*Lactiplantibacillus plantarum* PB22	8.16 ± 0.01^ab^
*Lactiplantibacillus pentosus* B307	8.19 ± 0.07^a^
*Lactiplantibacillus plantarum* B329	7.57 ± 0.35^c^
*Fructilactobacillus sanfranciscensis* B415	7.62 ± 0.14^c^
*Lactiplantibacillus pentosus* B506	8.12 ± 0.07^b^

The data are presented as means ± SDs. Significance at *P* < 0.05. By Tukey’s multiple range test, small letters show differences among the strains

### LAB bioconversion of oleuropein to hydroxytyrosol

Table 4 reports the effect of the LAB strains on the concentration of oleuropein and hydroxytyrosol over 28 days. Olive leaves naturally contain oleuropein, hydroxytyrosol (Bertelli et al. 2020) and other bioactive compounds. These compounds are water-soluble, meaning that when the ground leaves are submerged in the cultivation broth, they will naturally pass into solution.

**Table 4:** Oleuropein and Hydroxytyrosol content in the Olive leaf-containing broth fermented by the five LAB strains over 28 days

Analysis time (days)	Oleuropein (mg L^− 1^)					
	B415	B506	B307	PB22	B329	**Sign**
3	23.02 ± 26.70^A^	12.35 ± 1.27^aAB^	19.56 ± 17.24^A^	13.75 ± 0.52^cB^	18.13 ± 0.13^aB^	**
7	35.97 ± 34.26^A^	16.18 ± 0.54^cB^	28.64 ± 17.83^A^	17.09 ± 0.98^abB^	17.73 ± 0.35^bB^	**
14	20.38 ± 20.71^A^	10.61 ± 0.77^abB^	26.11 ± 6.52^A^	18.72 ± 0.88^aB^	15.44 ± 0.08^bB^	**
21	27.27 ± 9.77^A^	10.15 ± 0.56^bA^	27.91 ± 3.58^B^	14.26 ± 0.59^cB^	18.29 ± 0.08^aB^	**
28	28.84 ± 11.19^A^	10.79 ± 0.08^abB^	25.76 ± 2.62^A^	16.51 ± 0.37b^BC^	14.75 ± 0.21^cC^	**
**Sign**	n.s.	**	n.s.	**	**	
Analysis time (days)	Hydroxytyrosol (mg L^− 1^)					**Sign**
3	87.70 ± 7.75^AB^	112.19 ± 15.56^aA^	92.04 ± 4.35^aAB^	56.32 ± 1.22^aB^	60.42 ± 0.68^aB^	**
7	80.13 ± 12.61^AB^	100.49 ± 13.96^abA^	87.93 ± 0.81^abAB^	54.67 ± 0.93^aAB^	50.9 ± 0.93^bB^	*
14	52.09 ± 2.14	72.171 ± 1.16^bc^	59.14 ± 6.28^bc^	59.59 ± 9.72^a^	52.38 ± 1.13^b^	n.s.
21	50.06 ± 3.77^A^	66.24 ± 8.40^cB^	35.12 ± 4.24^cdBC^	34.65 ± 0.71^bBC^	32.5 ± 1.21^cC^	**
28	36.63 ± 2.96^AB^	52.15 ± 7.50^cA^	25.51 ± 1.97^dB^	33.31 ± 0.81^bB^	24.41 ± 0.93^dB^	**
**Sign**	n.s.	**	**	**	**	

The data are presented as means ± SDs. **Significance at *P* < 0.01; *Significance at *P* < 0.05; n.s. not significant. By Tukey’s multiple range test, small letters show differences during the time for all samples and capital letters show differences among the samples at the same time

For almost all strains the oleuropein content increased until the seventh day, when it decreased. This initial oleuropein increase could be due to its diffusion from the olive leaves into the cultivation broth. Two strains, *F. sanfranciscensis* B415 and *L. pentosus* B307, exhibited higher oleuropein content both in the first days of the experiment, and also maintained a higher concentration than the other strains until the end of the experiment.

Concerning hydroxytyrosol, 4 of the 5 LAB strains achieved their highest concentrations after 3 days, and the majority of them maintained it until day 7. As fermentation progressed, the hydroxytyrosol concentration of each strain decreased with more than 50% of it gone by day 28. The strains with the highest concentration of hydroxytyrosol after 3 days of fermentation were *F. sanfranciscensis* B415, *L. pentosus* B506, and *L. pentosus* B307; the best of these was *L. pentosus* B506. The hydroxytyrosol concentration does not arise only from oleuropein degradation by LAB but also from diffusion of the compound from the leaves into the cultivation broth. In the control (broth medium with ground olive leaves), the content of oleuropein was 10.50 mg L^− 1^ after 3 days and 6.05 mg L^− 1^ after 7 days, dropping to 0.81 mg L^− 1^ at later time points. Hydroxytyrosol was detected after 3 days and its content was 29.16 mg L^− 1^. These quantities are expected since the pH of the solution (5.94), the solvent used (water), and the temperature are not optimal for the extraction of these phenolic compounds. The drop in pH caused by the LAB strains improved the extraction of oleuropein and hydroxytyrosol from the leaves and their diffusion into the cultivation broth where the LAB strains then hydrolysed the oleuropein to hydroxytyrosol.

## Discussion

The β-glucosidase activity assay confirmed that some LAB strains are able to split the β-glycosidic bond, which is important for the bioconversion of oleuropein from olive leaves. The five strains studied here come from three species: *L. plantarum* and *L. pentosus* are common species in olive fermentation (Perpetuini et al. 2020) while *F. sanfranciscensis* is commonly found in sourdough (Martorana et al. 2018). To the best of our knowledge, this is the first report on the *β*-glu activity of *F. sanfranciscensis* and its possible use to convert oleuropein to hydroxytyrosol. This suggests that the research in this field should be widened to include LAB species not commonly associated with olives because useful strains may thereby be found.

The LAB strains significantly decreased the pH of the olive-leaf containing cultivation broth relative to the control. Similar results have been reported for LAB tested in modified MRS medium containing glucose and oleuropein (Iorizzo et al. 2016). This is expected because the metabolic activity of LAB strains is able to convert sugars and other nutritional components into acids (Ghabbour et al. 2016; Ilgaz et al. 2023).

The high LAB loads at the end of the experiment confirm that the olive leaves are able to support LAB growth (Iorizzo et al. 2016; Ilgaz et al. 2023; Sar et al. 2024). The strains, therefore, are able to grow and maintain high cell viability in the presence of low glucose and tolerate the olive leaf polyphenols. Indeed, previous researches have even reported that LAB growth can be enhanced by oleuropein and related compounds (Santos et al. 2012) and the optimal tolerance of *Lactobacillus* strains for phenolic compounds has been studied (Landete et al. 2008; Rodríguez et al. 2009). On the other hands, Gugel et al. (2024) have reported that olive leaves are not sufficient to support fermentation by *Lactobacillus casei* due to their high polyphenol content and low glucose content.

Regarding the conversion of oleuropein to hydroxytyrosol, Ilgaz et al. (2023) reported trends similar to our results. Likewise, Martínez-Navarro et al. (2021) observed that the hydroxytyrosol from oleuropein degradation exhibits an initial increase followed by a decrease due to the formation of derivative compounds. Hydroxytyrosol mainly undergoes degradation through oxidation and hydrolysis, producing compounds such as quinones and quinone dimers. The decrease of hydroxytyrosol could also be due to the formation of conjugated forms such as the 3,4-(dihydroxyphenyl)ethyl ester of elenolic acid, verbascoside, and hydroxytyrosol glycosides (Bianco et al. 2006; Fernández-Bolaños et al. 2008; Wang et al. 2025b).

Despite the inefficient extraction conditions, our results show an increase in the content of hydroxytyrosol – at least in the first part of the fermentation – which is due partly to its diffusion from the ground leaves aided by the acidic pH (Yateem et al. 2014; Rubio-Senent et al. 2017), a result of the fermentation, and partly to the hydrolysis of oleuropein due to the β-glu activity of the LAB strains.

Usually, the extraction of phenolic compounds from olive leaves is carried out using organic solvents, assisted by microwaves and ultrasound (Yateem et al. 2014; Cifá et al. 2018; Martínez-Navarro et al. 2021; Papageorgiou et al. 2022). These methods involve energy consumption and disposal of the solvents pose a problem. For use in a green economy, we have studied and propose a simple, eco-friendly and cost-effective laboratory-scale method based on the use of LAB. This method does not use a chemical solvent but a cultivation broth for LAB strains with a reduced glucose content (a high glucose content impairs the LAB strains’ β-glu activity) (Marsilio and Lanza 1998). In the LAB fermentation, carried out at a mild temperature, the strains have a double role: they generate a low pH, useful for the extraction of polyphenol compounds from the olive leaves, and they hydrolyse the oleuropein to hydroxytyrosol. The current conditions are probably not optimal, however, and to improve the present method in order to obtain a higher hydroxytyrosol yield further studies are needed. Two possible research lines appear interesting to explore: modifying the LAB cultivation broth, for example by testing different concentrations of NaCl (which has been reported to be useful in the conversion of oleuropein to hydroxytyrosol (Bouaziz et al. 2010; Ghabbour and Rokni 2020; Ilgaz et al. 2023)), and testing different LAB strains in co-fermentation or sequential fermentation.

Given that oleuropein occurs in many genera belonging to the *Oleaceae* family such as *Fraxinus excelsior*, *Fraxinus angustifolia*, *Fraxinus chinensis* and *Fraxinus mandshurica* var. *japonica*, *Syringa josikaea*, *Ligustrum ovalifolium* and *Ligustrum vulgare*, *Jasminum polyanthum* and *Osmanthus asiaticus* (Soler-Rivas et al. 2000), the present method could be useful for recycling not only olive tree pruning wastes but also those of other ornamental plants, thereby obtaining valuable compounds.

Since hydroxytyrosol has positive effects on human health, it has great potential to be widely used in industrial (cosmetics, food) and pharmaceutical fields. Many challenges remain, including maximizing the yield according to the method used, identifying novel hydroxytyrosol derivatives possessing higher bioactivity, optimizing the production scalability, and increasing the bioavailability by proposing novel carriers (example: lypophilic) or novel delivery technologies such as nanoparticles and active packaging.

## Conclusion

Improper disposal of plant pruning wastes remains a global concern with implications for both environmental pollution and farm disposal costs. The focus of this study was the microbiological treatment of leaves from *Olea europaea* L. in order to obtain valuable bioactive compounds such as hydroxytyrosol. Three LAB strains – *F. sanfranciscensis* B415, *L. pentosus* B506, and *L. pentosus* B307 – were the best strains for converting olive leaf oleuropein into hydroxytyrosol after 3 days of fermentation. The method developed in this study is easy and eco-friendly since it involves no chemical solvents and, from the perspective of a sustainable agriculture and circular economy, it may prove useful for recycling the pruning wastes of olive trees or other plants rich in oleuropein.

## Data Availability

No datasets were generated or analysed during the current study.
